# Epidemiology of COVID-19 after Emergence of SARS-CoV-2 Gamma Variant, Brazilian Amazon, 2020–2021

**DOI:** 10.3201/eid2803.211993

**Published:** 2022-03

**Authors:** Vanessa C. Nicolete, Priscila T. Rodrigues, Anderson R.J. Fernandes, Rodrigo M. Corder, Juliana Tonini, Lewis F. Buss, Flávia C. Sales, Nuno R. Faria, Ester C. Sabino, Marcia C. Castro, Marcelo U. Ferreira

**Affiliations:** University of São Paulo, São Paulo, Brazil (V.C. Nicolete, P.T. Rodrigues, A.R.J. Fernandes, R.M. Corder, J. Tonini, L.F. Buss, F.C. Sales, N.R. Faria, E.C. Sabino, M.U. Ferreira);; Imperial College London, London, UK (N.R. Faria);; University of Oxford, Oxford, UK (N.R. Faria);; Harvard T.H. Chan School of Public Health, Boston, Massachusetts, USA (M.C. Castro)

**Keywords:** COVID-19, 2019 novel coronavirus disease, coronavirus disease, severe acute respiratory syndrome coronavirus 2, SARS-CoV-2, viruses, respiratory infections, zoonoses, Gamma variant, seroprevalence, Amazon, Brazil

## Abstract

The severe acute respiratory syndrome coronavirus 2 (SARS-CoV-2) Gamma variant has been hypothesized to cause more severe illness than previous variants, especially in children. Successive SARS-CoV-2 IgG serosurveys in the Brazilian Amazon showed that age-specific attack rates and proportions of symptomatic SARS-CoV-2 infections were similar before and after Gamma variant emergence.

The novel severe acute respiratory syndrome coronavirus 2 (SARS-CoV-2) Gamma (P.1) variant emerged in November 2020 and drove the second wave of coronavirus disease (COVID-19) in Brazil. Emergence of this variant in Manaus, the largest city in the Brazilian Amazon, was followed by a dramatic upsurge in deaths across the region in early 2021 (*1*,*2*). Gamma harbors amino acid substitutions in the angiotensin-converting enzyme 2 receptor–binding domain of the spike protein, which are thought to enhance host cell infectivity (*3*). This variant may be 1.7–2.4 times more transmissible than previously circulating variant lineages of SARS-CoV-2 (*3*) and can evade antibodies elicited by prior infection or vaccination (*4*,*5*).

During the first COVID-19 epidemic wave, symptoms were half as likely to develop in young children with SARS-CoV-2 infection than in adults >30 years of age, according to an ongoing population-based cohort study in the Brazilian Amazon (*6*). However, patients hospitalized for COVID-19 during the Gamma-dominated second wave in Brazil tended to be younger and more likely to die (*7*), suggesting that Gamma might cause more severe illness, especially in children (*8*). To determine the epidemiology of COVID-19 after emergence of SARS-CoV-2, we compared age-specific COVID-19 attack rates and proportions of symptomatic SARS-CoV-2 infections in the cohort before and after spread of the Gamma variant in the Amazon. The National Committee of Ethics in Research, Ministry of Health of Brazil (CAAE no. 30481820.3.0000.5467), approved the study protocol. Written informed consent was obtained from study participants or their parents/guardians.

## The Study

**Figure 1:**
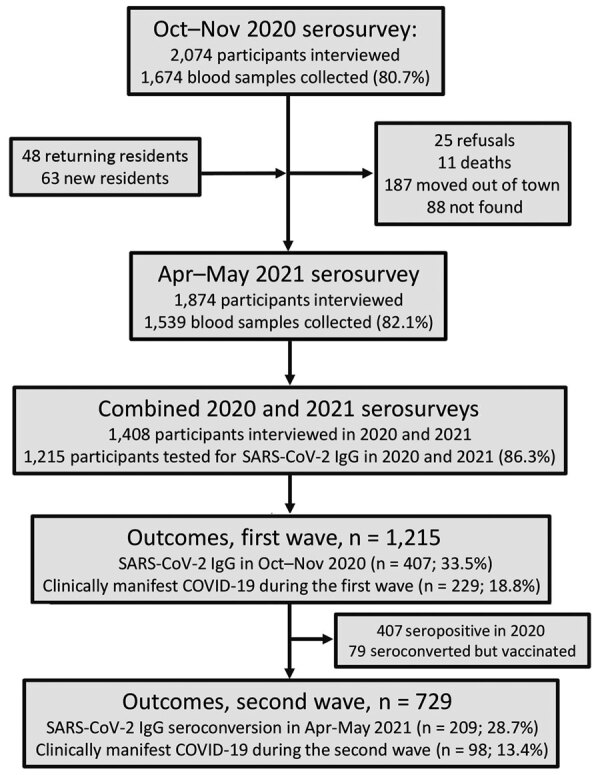
Study of epidemiology of COVID-19 after emergence of SARS-CoV-2 Gamma variant, Brazilian Amazon, 2020–2021. COVID-19, coronavirus disease; SARS-CoV-2, severe acute respiratory syndrome coronavirus 2.

**Figure 2:**
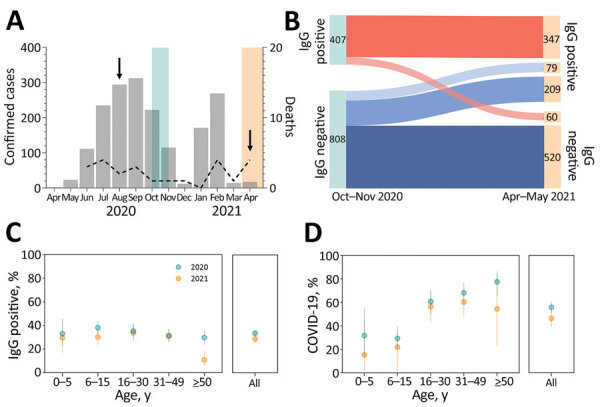
Severe acute respiratory syndrome coronavirus 2 (SARS-CoV-2) infection and clinically apparent coronavirus disease (COVID-19) during the first and second epidemic waves, Brazilian Amazon, 2020–2021. A) Monthly cases (bars) and COVID-19–associated deaths (dashed line) notified in the municipality of Mâncio Lima, Brazil, during April 2020–April 2021. Light blue shading represents serosurveys conducted during October–November 2020; light peach shading represents serosurveys conducted during April–May 2021; arrows indicate dates of SARS-CoV-2 isolate collection for genomic surveillance. B) Distribution of study participants (n = 1,215) according to SARS-CoV-2 IgG detected in each serosurvey. The 288 for whom IgG seroconverted during April–May 2021 includes 79 vaccinated persons (light blue), who were not considered when estimating rates of seroconversion resulting from natural SARS-CoV-2 infection. C) Age-specific percentages of persons positive for SARS-CoV-2 IgG at the end of the first wave (October–November 2020; light blue dots) and of IgG seroconversions among initially seronegative persons by the second wave (April–May 2021; light peach dots). Error bars indicate 95% CIs. D) Age-specific percentages of SARS-CoV-2 infections that led to clinically apparent COVID-19 during the first wave (light blue dots) and second wave (light peach dots). Denominators correspond to the number of participants with serologic evidence of SARS-CoV-2 infection during the period. Error bars indicate 95% CIs.

Follow-up of the Mâncio Lima cohort in the Brazilian Amazon (https://www.niaid.nih.gov/research/amazonian-international-center-excellence-malaria-research), which accounts for 20% of the town’s 9,000 residents, started in April 2018 (Figure 1; Appendix Methods). The first COVID-19 case in Mâncio Lima was notified on April 29, 2020; as of April 30, 2021, a total of 1,797 laboratory-confirmed infections and 24 deaths were recorded (Figure 2, panel A).

We estimated overall and age-specific SARS-CoV-2 attack rates and the proportion of infections leading to clinically apparent COVID-19 during the first and second epidemic waves in Mâncio Lima. We tested 1,215 cohort participants, <1 to 93 (median 29) years of age, for IgG to the subdomain S1 of the SARS-CoV-2 spike protein (Euroimmun ELISA, EI 2606–9601 G; PerkinElmer, https://www.perkinelmer) during October–November 2020 (*6*) and April–May 2021 (Figure 2, panel A). We obtained information about sociodemographics, COVID-19 exposures, and history of recent illness and vaccination. As a simplifying assumption, we considered seropositive participants in 2020 to not be at risk for reinfection during the second wave, but we attempted to identify instances of antibody boosting, which might represent reinfection. We excluded IgG seroconversions in COVID-19–vaccinated participants because our serologic testing does not distinguish natural infection from vaccination (Figure 1; Appendix).

We collected nasopharyngeal specimens from patients seeking COVID-19 testing in August 2020 and April 2021 to genetically characterize local SARS-CoV-2 isolates (Appendix Methods) with nanopore sequencing on a MinION platform (Oxford Nanopore, https://nanoporetech.com), using the ARTIC V3 protocol (J.R. Tyson et al., unpub. data, https://www.biorxiv.org/content/10.1101/2020.09.04.283077v1). We used Pangolin version 3.1.5 (*9*) to classify SARS-CoV-2 lineages. The 14 isolates from August 2020 (*6*) were assigned to the B.1.1.33 lineage (*10*), and all 11 SARS-CoV-2 isolates from April 2021 were the Gamma variant (Appendix Table 1; GISAID accession nos. EPI_ISL_2987666–74, EPI_ISL_2988699, and EPI_ISL_2988700), which dominated the second wave.

Outcomes were 1) SARS-CoV-2 IgG positivity (2020 survey) or IgG seroconversion in the absence of COVID-19 vaccination (2021 survey), as proxies of SARS-CoV-2 infection, to estimate attack rates during the first and second waves, respectively; and 2) presence of >1 sign/symptom—new or increased fever, cough, shortness of breath, chills, muscle pain, loss of taste or smell, sore throat, diarrhea, or vomiting—within the past 6 months (*6*), self-reported by participants with serologic evidence of SARS-CoV-2 infection, as a proxy of clinically apparent COVID-19. We excluded 79 persons vaccinated for COVID-19 who seroconverted (Appendix Results, Figure 2). For each outcome, we used Stata 15.1 (StataCorp LLC, https://www.stata.com) to estimate adjusted relative risks, along with 95% CIs, and used mixed-effects Poisson regression models with random effects at the household level and robust variance (Appendix Methods). Statistical significance was defined at the 5% level.

Most Mâncio Lima residents (54.2%, 95% CI 51.3%–57.1%) demonstrated serologic evidence of SARS-CoV-2 infection at the end of the study (first and second waves combined); sensitivity/specificity-adjusted prevalence (Appendix) was 65.0% (95% highest density interval [95% HDI] 58.5%–73.9%). This finding is consistent with the high COVID-19 attack rates observed in population-based studies in the Amazon (*11*–*13*). One third of study participants (33.5%, 407/1,215) were seropositive at the end of the first wave, and adjusted seroprevalence was 38.9% (95% HDI 33.2%–44.8%). Ten (0.8%) participants reported having been hospitalized between April 2020 and the first survey (blood sampling and questionnaire administration; missing information for 4 study participants), but we did not explicitly ask whether the cause of hospital admission was COVID-19; only 4 of 10 patients who reported hospital admissions (28, 66, 58, and 68 years of age) were seropositive. Among 729 initially seronegative participants, 209 (28.7%) seroconverted (adjusted prevalence 32.7%, 95% HDI 26.7%–38.9%) by the second visit but were not vaccinated (Figure 2, panel B; Appendix Results, Figure 1). We specifically asked for COVID-19–associated hospitalizations, and 7 (0.6%) participants (32, 32, 67, 58, 71, 3, and 81 years of age) reported hospital admissions during the second wave.

Of the 407 participants who were seropositive at the time of the first survey, 60 (14.7%, 95% CI 11.4%–18.6%) became negative (seroreverted) by April–May 2021 (Figure 2, panel B). Of the 347 persistently seropositive participants, antibody reactivity index increased by >2-fold for 46 (13.3%) by April–May 2021 (Appendix Figure 3); 18 (5.2%) of the 347 were not vaccinated and therefore may have experienced reinfection during the second wave. Only 4 (22.2%) of the 18 participants with possible reinfection reported clinical manifestations (Appendix Results); the rest were asymptomatic.

At the end of the first and second epidemic waves, antibody positivity and seroconversion rates were similar across age groups, except for adults >50 years of age, among whom there were proportionally fewer infections in the second wave than in the first wave (Figure 2, panel C; Appendix Table 2). A smaller proportion of SARS-CoV-2–infected persons were symptomatic during the second wave (46.9% [95% CI 40.0%–53.9%]) than during the first wave (56.3% [95% CI, 51.3%–61.1%]; p = 0.034 by Yates-corrected χ^2^ test) (Figure 2, panel D).

During the second wave, risk for SARS-CoV-2 infection was similar for young children and adults, but risk for symptomatic COVID-19 was lower for children than for adults. Similar trends were observed during the first wave (Appendix Tables 2, 3). After infection, clinical signs/symptoms were significantly less likely to develop in young children than in adults during both epidemic waves (Figure 2, panel D), even after we adjusted for potential confounders by using multiple Poisson regression (Appendix Table 4).

## Conclusions

In this Brazilian Amazon cohort, we found no evidence that SARS-CoV-2 infections acquired during the second epidemic wave, dominated by the Gamma variant, produced more symptomatic illness than infections acquired during the first wave. Of note, symptomatic infections did not affect young children disproportionally more during the second wave. The explosive increase in illness and death in the Amazon during the second COVID-19 wave most likely reflects the rapid spread of a highly transmissible variant of concern, the regional and federal government’s failure to enforce nonpharmaceutical interventions to curb community transmission of SARS-CoV-2, and limited availability of intensive care beds to cope with severe cases of COVID-19 (*14*).

Appendix Supplementary methods and results for study of epidemiology of COVID-19 after emergence of SARS-CoV-2 Gamma variant, Brazilian Amazon, 2020–2021.
