# Supplementary material for: Epidemiology of COVID-19 after Emergence of SARS-CoV-2 Gamma Variant, Brazilian Amazon, 2020–2021
Source: Emerg Infect Dis. 2022 Mar;28(3):709–12. doi: 10.3201/eid2803.211993 (PMC8888227; doi:10.3201/eid2803.211993)
Supplement: Appendix — Supplementary methods and results for study of epidemiology of COVID-19 after emergence of SARS-CoV-2 Gamma variant, Brazilian Amazon, 2020–2021. [file 21-1993-Techapp-s1.pdf]

Epidemiology of COVID-19 after Emergence of SARS-CoV-2 Gamma Variant, Brazilian Amazon, 2020–2021

Appendix

Supplementary Methods

The Mâncio Lima cohort study

The Mâncio Lima cohort study is part of the National Institutes of Health (NIH)-funded Amazonian International Center of Excellence for Malaria Research network, with the overall aim of investigating malaria epidemiology, vector biology and ecology, diagnostics, transmission biology, and clinical pathogenesis (https://www.niaid.nih.gov/research/amazonian-international-center-excellence-malaria-research). This population-based open cohort study was set up in 2018 to investigate a wide range of biologic and sociodemographic factors that drive malaria endemicity in the main urban transmission hotspot of Amazonian Brazil. The original study has since expanded to include SARS-CoV-2 antibody measurements (*1*).

We carried out a baseline population census in the town of Mâncio Lima between November 2015 and April 2016. We enumerated 9,124 permanent residents in the urban area, with ages ranging between <1 month and 105 years, distributed into 2,329 households (*2*). The cohort study participants are members of randomly chosen urban households in Mâncio Lima. We used simple probability sampling to draw 534 households from the list of those enumerated during the baseline census survey. We initially allowed for up to 2.9% non-localized or empty houses and refusals and aimed to enroll at least 20% of all households in the town. Because the target sample size was not reached during the first visit, we used a list of randomly chosen substitute households during the second visit to replace households that had declined participation or were not located (*1*). Because this cohort was set-up to evaluate a wide range of exposures and malaria-related outcomes in the same population, no formal a-priori sample size and power calculations were made.

The first study visit, between April and May 2018, targeted the 534 households drawn from the census listings; 1,391 residents from 354 households were located and agreed to participate. To achieve the desired sample size, 147 substitute households were randomly selected and approached during the second visit, in October-November 2018. The ongoing cohort is dynamic and new residents joining the household (those who moved in or were born between study visits) are enrolled during the follow-up visits. Study participants leaving the sampled households are retained in the cohort as long as they can be located by the field team and their new residences, which are labeled as new households, are situated in the urban area of Mâncio Lima. Six sequential house-to-house visits were carried out so far.

The median age of participants in the Mâncio Lima cohort study is 22 years (range, <1 to 103 years), with 51.3% of females. Among study participants ≥10 years of age, the literacy rate is 91.8%. Only 9.9% adult participants have a formal job. Most (80.0%) of study participants are supported by the Federal conditional cash transfer program called "Bolsa Família," a proxy of poverty.

Data and samples analyzed in the present study were obtained during serosurveys carried out in October-November 2020 and April-May 2021. The 2020 survey comprised 2,074 subjects distributed into 567 households, while the 2021 survey comprised 1,874 subjects distributed into 540 households. Overall, 1,408 individuals participated in both surveys; 1,215 (86.3%) of them (56.3% females) were tested for anti-SARS-CoV-2 antibodies on both occasions (main text, Figure 1). Compared to untested individuals, participants with antibody data in 2021 were significantly older (mean, 31.7 versus 26.4 years; $p < 0.001$, *t*-test), more likely to be females (56.3% versus 44.1%; $p < 0.001$, χ^2 test) and less likely to report at least one overnight stay outside Mâncio Lima within the past 6 months (29.1% versus 41.3%; $p < 0.001$, χ^2 test). No significant differences were observed regarding other covariates of interest.

SARS-CoV-2 IgG antibody detection

We tested 1,215 paired plasma samples, obtained from the same study participants at a 6-month interval during the 2020 and 2021 surveys, for anti-SARS-CoV-2 IgG with a semiquantitative ELISA that uses the recombinant subdomain S1 of the Spike protein as antibody-capture antigen (EI 2606–9601 G; Euroimmun) (*3*). The assay has a sensitivity of

82.5% to 93.3% and specificity of 98.0% to 98.5% (*4*,*5*). Results from the 2020 survey were previously published (*6*).

We used a quantitative ELISA to investigate changes in specific antibody concentrations in selected paired plasma samples. To this end, we added to each microplate a standard curve with a serially diluted pool of 10 strongly positive plasmas (eight dilutions from 1:25 to 1:1:3,200). We defined that the pool had an antibody concentration of 100 arbitrary units (AU) at a 1:25 dilution. Antibody concentrations in test samples were interpolated using a four-parameter logistic regression model. Samples were tested at a 1:100 dilution and those with absorbance values outside the range of the standard curve (i.e., absorbances >3.363 or <0.527) were assigned antibody concentrations of 110 AU and 0.7 AU, respectively.

SARS-Cov-2 detection

To characterize SARS-CoV-2 lineages circulating during the first and second waves, we obtained two nasopharyngeal swab samples from 49 consecutive symptomatic patients (age range, 3–77 years) seeking COVID-19 testing in Mâncio Lima in August 2020 and again from 49 patients (age range, 4–86 years) in April 2021. Results obtained with the samples collected in 2020 were published elsewhere (*6*).

One swab collected between 21 and 29 April 2021 was used for point-of-care antigen-based diagnosis (ECO F COVID-19 Ag test FA0054; Ecodiagnostica, Corinto, Brazil) and the other was preserved in RNA/DNA Shield (Zymo Research, Irvine, CA) for RNA extraction. Template RNA was prepared using QIAamp Viral RNA mini kits (Qiagen, Hilden, Germany). We tested antigen-positive samples for SARS-CoV-2 RNA by reverse transcription PCR by using the China CDC protocol that targets the ORF1ab and N genes (XGEN Master COVID-19 kit, Mobius Life Science, Pinhais, Brazil). Target amplification was carried out as described (*7*).

SARS-CoV-2 genome sequencing

We selected 15 samples with cycle threshold <30 for whole-genome sequencing as part of a countrywide SARS-CoV-2 genomic surveillance project (*8*). Template RNA was converted to cDNA using the Protoscript II First Strand cDNA synthesis Kit (New England Biolabs, Cambridge, MA) and random hexamers. Whole-genome amplification was performed with multiplex PCR amplification using the SARS-CoV-2 primer scheme (V1 to V3) and Q5 High-Fidelity DNA polymerase (New England Biolabs, UK), by using ARTIC protocol

(https://www.protocols.io/view/ncov-2019-sequencing-protocol-bbmuik6w?version_warning = no). PCR products were cleaned-up using AmpureXP purification beads (Beckman Coulter, High Wycombe, UK) and quantified using the Qubit dsDNA High Sensitivity assay on the Qubit 3.0 instrument (Life Technologies, Thermo Fischer Scientific, USA). Amplicons from each sample were normalized and pooled in an equimolar fashion and barcoded using the EXP-NBD104 (1–12) and EXP-NBD114 (13–24) Native Barcoding Kits (Oxford Nanopore Technologies, UK). Concentrations of double-stranded DNA for the library-negative controls were below detection levels, indicating no contamination.

Nanopore sequencing on the MinION platform (Oxford Nanopore, Oxford, UK) was carried out libraries were generated using the SQK-LSK109 Kit (Oxford Nanopore) and were loaded onto an R9.4.1 flow-cell (Oxford Nanopore). RAMPART software from the ARTIC Network (https://artic.network/ncov-2019/ncov2019-using-rampart.html) was used to monitor the sequencing run in real time to estimate the coverage depth (target, 200×). With the Guppy software version 4.4.0 (Oxford Nanopore Technologies), fastq files were base-called, demultiplexed, and trimmed. Sequencing data were subjected to sequence quality controls and the consensus genomes were obtained by the mapping of fastq files to Wuhan-Hu 1 reference genome (GenBank Accession Number MN908947).

Assembled sequences of 11 isolates (out of 15) yielded at least 50% coverage of the SARS-CoV-2 genome, with at least 20× depth. Lineages were classified using the Pangolin COVID-19 Lineage Assigner software tool (http://pangolin.cog-uk.io/) and phylogenetic analysis using complete reference genomes. Sequencing statistics and lineage assignment information are provided in Appendix Table 1.

Estimating COVID-19 attack rates

We used IgG positivity during the first survey (October-November 2020) as a proxy of SARS-CoV-2 infection during the first wave. The crude antibody prevalence (%) in October-November 2020, a proxy of the COVID-19 attack rate between April and November 2020, was calculated as number of IgG positive persons (n = 407) divided by the number of participants tested (n = 1215) × 100, with exact binomial 95% confidence intervals. We used IgG seroconversion detected in the second survey as a proxy of SARS-CoV-2 infection during the second wave. The attack rate between surveys was calculated as the number of IgG

seroconversions in April-May 2021 in participants who had not been vaccinated (n = 209) divided by the number of participants who were IgG-negative during the first survey (n = 729). To this end, we excluded from both the numerator and the denominator the 79 participants who seroconverted but had been vaccinated until the date of the latest study visit (April-May 2021), as they might have developed SARS-CoV-2 antibodies upon vaccination rather than natural infection. COVID-19 vaccination in Mâncio Lima started in February 2021, with the inactivated vaccine CoronaVac (Sinovac Life Sciences, China) and the adenoviral-vectored vaccine ChAdOx1 nCoV-19 (Oxford University–AstraZeneca, UK). The initial target populations for COVID-19 vaccination were health professionals and persons >60 years of age.

To estimate the overall attack rate during the whole study period, we considered all participants with IgG antibodies detected in the first survey (n = 407) and all unvaccinated IgG seroconversions detected in the second survey (n = 209), giving a total of 616 cohort participants with serologic evidence of SARS-CoV-2 infection in the numerator. The denominator was 1215 - 79 = 1136 participants, as the 79 vaccinated subjects were excluded.

As well as crude antibody prevalence, we also present sensitivity and specificity adjusted prevalence estimates. We used a Bayesian framework that propagates uncertainty in the sensitivity and specificity estimates of the test (*9*). We used the validation data from Naaber et al. (*4*) in which 80 out of 97 PCR-confirmed SARS-CoV-2 cases tested positive on the Euroimmun IgG assay, and 98 out of 100 known negative samples tested negative. The point estimate along with the 95% highest density interval are presented.

Data analysis

Data were transferred from tablets programmed with REDCap (*10*) to STATA 15.1 (StataCorp, College Station, TX) for analysis. Six multiple Poisson regression models (*11*) were built to identify factors associated with each binary outcome: (i) SARS-CoV-2 infection during the first wave (Appendix Table 2); (ii) clinically apparent COVID-19 during the first wave (Appendix Table 3), (iii) clinically apparent COVID-19 upon serologically documented SARS-CoV-2 infection during the first wave (Appendix Table 4); (iv) SARS-CoV-2 infection (using IgG seroconversion as a proxy) during the second wave, among participants who were seronegative during the first survey (October-November 2020; Appendix Table 2); (v) clinically apparent COVID-19 during the second wave among participants who were seronegative during

the first survey (October-November 2020; Appendix Table 3); and (vi) clinically apparent COVID-19 during the second wave among participants who were seronegative during the first survey (October-November 2020) and seroconverted by April-May 2021 (Appendix Table 4).

Note that models (ii) and (iii), as well as models (v) and (vi), have the same numerators (numbers of individuals with clinically apparent COVID-19 symptoms plus positive serology) but the denominators are different. Denominators in models (ii) and (v) are the entire susceptible population (n = 1027 participants with complete information in the first wave [model ii] and n = 729 with complete information in the second wave [model v]). Therefore, models (ii) and (v) explore the risk factors for serologically proven, symptomatic COVID-19 during the first and second waves in the entire study population. In contrast, models (iii) and (vi) explore the risk factors for symptomatic COVID-19 among individuals with serologically proven SARS-CoV-2 infection during the first and second waves. Denominators are the total number of seropositive participants at the end of the first wave (n = 359 [model iii]) and total number of seroconverters during the second wave (n = 209 [model vi]).

Because study participants are nested into households, which introduces dependency among observations, for each outcome we built mixed-effects Poisson regression models with random effects at the household level and robust variance. Individual covariates were age in October-November 2020 (categorical variable), sex (female versus male), laboratory-confirmed malaria within the past 12 months (no versus yes), overnight stay(s) away from Mâncio Lima within the past 12 months (no versus yes), and DENV seropositivity in the previous serosurvey (either October-November 2019 or October-November 2020; no versus yes). Household covariates were wealth index quintiles (*6*) and household size. Age, sex, and covariates associated with the outcome at a significance level <20% in unadjusted analysis were retained in multiple Poisson regression models. Participants with missing values were excluded from the adjusted models. Statistical significance was defined at the 5% level; relative risk (RR) estimates are provided along with 95% confidence intervals (CIs) to quantify the influence of each predictor on the outcome, while controlling for all other covariates (*11*).

Supplementary Results

SARS-CoV-2 attack rates during the first and second waves

We observed a higher attack rate between April and November 2020 (33.5%; 95% CI, 30.8%–36.2%) compared with that between November 2020 and April 2021 (28.7%; 95% CI, 25.4%–32.1%). However, differences in attack rate over time must not be overinterpreted because populations at risk are not entirely comparable during the first and second waves. We argue that SARS-CoV-2 has affected disproportionately the most exposed and most susceptible persons in our heterogeneous cohort population. High-risk participants were infected first and developed specific antibodies more rapidly; as a consequence, SARS-CoV-2 transmission during the first epidemic wave may have selectively removed high-risk individuals from the pool of seronegatives (*12*). Moreover, some high-risk population strata (health professionals and persons >60 years of age) were selectively vaccinated (see below). A proportionally larger fraction of individuals who remained seronegative after the first wave is expected to be either unexposed or little susceptible to SARS-CoV-2 infection, limiting virus spread during the second wave. This concept is illustrated in Appendix Figure 1.

IgG seroconversion after COVID-19 vaccination

Overall, 160 (13.2%; 95% CI, 11.4%–15.2%) study participants reported having been partially or fully vaccinated against COVID-19 until the date of the latest survey (April-May 2021). The locally available vaccines were the CoronaVac vaccine (administered to 64 participants; 40.0% of the vaccinees) and the ChAdOx1 nCoV-19 vaccine (administered to 89 participants; 55.6% of the vaccinees). Seven persons (4.4%) did not report the vaccine received. Most (78.8%) vaccinees were ≥60 years of age and 11.3% were health professionals. Among vaccinees, 94 (58.8%) had received a single vaccine dose and 59 (36.9%) had received both doses at the time of the latest survey; seven did not report the number of doses administered.

Seroconversion rates measured in April-May 2021 were much higher among vaccinees than in the general population. There were 107 study participants who were SARS-CoV-2 seronegative in 2020 and received one or more doses of a COVID-19 vaccine in 2021. Of them, 79 seroconverted (73.8%; 95% CI, 64.5%–81.4%). Considering participants with known vaccine administered, seroconversion rates were similar for recipients of the CoronaVac vaccine (73.7%; 95% CI, 56.8%–85.6%, n = 38) and the ChAdOx1 nCoV-19 vaccine (76.6%; 95% CI, 64.3%–

85.5%, n = 64) (Appendix Figure 2). Estimated seroconversion rates were 72.5% (95% CI, 60.4%–81.9%; n = 94) for a single vaccine dose and 75.6% (95% CI, 58.6%–87.2%; n = 59) for two vaccine doses.

Increased antibody concentration in paired sequential plasma samples

The majority of study participants with SARS-CoV-2 IgG antibodies detected in October-November 2020 remained seropositive in April-May 2021 (347/407, 85.3%). Appendix Figure 3 shows that 46 of those persistently seropositive persons had a substantial increase in antibody reactivity (>2-fold increase in reactivity index) between surveys, consistent with a boosting antibody response due to a new infection or vaccination. As shown in Appendix Figure 4, 28 (70.9%) of the 46 participants with increased antibody levels reported having been vaccinated after the first survey and 18 (39.1%) remained unvaccinated. We conclude that 18 participants (5.2%), out of 347 persons with persisting SARS-CoV-2 antibodies, had an antibody boosting consistent with SARS-CoV-2 reinfection during the second wave. Their ages range between 1 and 75 years (mean, 25.4 years). Interestingly, only 4 (22.2%) of them reported clinical symptoms suggestive of COVID-19 since November 2020. By using a quantitative ELISA, we estimate that antibody concentrations increased, on average, 8.5-fold among the study participants with serologic evidence of a new infection during the second epidemic wave (Appendix Figure 4).

Predictors of SARS-CoV-2 infection and clinically apparent COVID-19 during the first and second epidemic waves

Participants living in crowded households (≥7 people) were at increased risk of SARS-CoV-2 infection during both the first and second waves. Female sex and affluence (highest wealth index quintile) were significantly associated with an increased risk of infection only during the first wave, while age ≥50 years predicted a decreased risk of infection only during the second wave (Appendix Table 2).

We considered the following self-reported symptoms to define clinically apparent COVID-19: new or increased fever, cough, shortness of breath, chills, muscle pain, loss of taste or smell, sore throat, diarrhea, or vomiting within the past 6 months. Children ≤5 years of age tended to be at lower risk of clinically apparent COVID-19 than adults during both waves, although statistical significance was not reached in most comparisons (Appendix Table 3). In

addition, affluence and household crowding were associated with a significantly increased risk of clinically apparent COVID-19 during the first wave (Appendix Table 3). The previously described association between a positive DENV IgG serology and subsequent risk of clinically apparent COVID-19 (*6*) reached statistical significance only during the first wave.

We next used multiple Poisson regression models to identify the predictors of clinically apparent COVID-19 among study participants with serologically proven SARS-CoV-2 infection during the first wave (IgG seropositivity in October-November 2020; n = 359 after excluding persons with missing information) and the second wave (IgG seroconversion in April-May 2021; n = 209). We further confirm that, during both waves, study participants >15 years of age tended to be similarly more likely to develop symptoms, once infected with SARS-COV-2, than young children (Appendix Table 4).

Some of the symptoms used to define clinically apparent COVID-19 may be found in other locally prevalent infectious diseases, such as malaria, dengue and common upper your upper respiratory tract. Malaria is unlikely to be a confounder in this population (Appendix Tables 2 and 4; see also reference *6*), but the annual dengue transmission season (November to April) overlapped with the second SARS-CoV-2 wave in 2020–21. As a consequence, the proportion of symptomatic SARS-CoV-2 infections during the second wave may have been slightly overestimated due to dengue symptoms reported by our study participants.

COVID-19 severity during the first and second waves

We found no evidence that SARS-CoV-2 infections acquired during the second epidemic wave, dominated by the Gamma variant, are more likely to be symptomatic in our study population. In contrast, a recent study has shown that, among people hospitalized in Brazil due to COVID-19, the median age of patients decreased (63 years vs 59 years), with a relative increase of 18% in the proportion of patients younger than 60 years during the second wave (the period from week 44 in 2020 to week 21 in 2021) compared with the first wave (weeks 8 to 43 in 2020). The in-hospital mortality increased from 33·1% to 40·6% during the same period (*13*).

There are several factors that may have contributed to these results. First, we can hypothesize that individuals at increased risk of infection may have been preferentially infected during the first wave. In addition, individuals >60 years were among the early targets of mass vaccination campaigns. As individuals who have been vaccinated or experienced natural

infection are less likely to develop severe disease once (re)infected during the second wave, some differences in age-specific hospitalization rates are expected. In other works, individuals at high risk (including those vaccinated in early 2021) were selectively removed from the "susceptible pool" (Appendix Figure 1).

Second, individuals admitted to overwhelmed hospitals during the second epidemic wave, which was particularly intense in Brazil, are likely to have, on average, a more severe disease than those admitted during the first wave. The number of hospital admissions mirrors the number of available beds, not necessarily the number of patients who required intensive care. Patients with more threatening clinical conditions are expected to be selectively admitted when few hospital beds are available.

References

1. Johansen IC, Rodrigues PT, Tonini J, Vinetz J, Castro MC, Ferreira MU. Cohort profile: the Mâncio Lima cohort study of urban malaria in Amazonian Brazil. BMJ Open. 2021;11:e048073. PubMed https://doi.org/10.1136/bmjopen-2020-048073

2. Corder RM, Paula GA, Pincelli A, Ferreira MU. Statistical modeling of surveillance data to identify correlates of urban malaria risk: A population-based study in the Amazon Basin. PLoS One. 2019;14:e0220980. PubMed https://doi.org/10.1371/journal.pone.0220980

3. Okba NMA, Müller MA, Li W, Wang C, GeurtsvanKessel CH, Corman VM, et al. Severe acute respiratory syndrome coronavirus 2-specific antibody responses in coronavirus disease patients. Emerg Infect Dis. 2020;26:1478–88. PubMed https://doi.org/10.3201/eid2607.200841

4. Naaber P, Hunt K, Pesukova J, Haljasmägi L, Rumm P, Peterson P, et al. Evaluation of SARS-CoV-2 IgG antibody response in PCR positive patients: Comparison of nine tests in relation to clinical data. PLoS One. 2020;15:e0237548. PubMed https://doi.org/10.1371/journal.pone.0237548

5. Tuaillon E, Bolloré K, Pisoni A, Debiesse S, Renault C, Marie S, et al. Detection of SARS-CoV-2 antibodies using commercial assays and seroconversion patterns in hospitalized patients. J Infect. 2020;81:e39–45. PubMed https://doi.org/10.1016/j.jinf.2020.05.077

6. Nicolete VC, Rodrigues PT, Johansen IC, et al. Interacting epidemics in Amazonian Brazil: prior dengue infection associated with increased COVID-19 risk in a population-based cohort study. Clin Infect Dis. 2021;73:2045–54. PubMed https://doi.org/10.1093/cid/ciab410

7. China CDC. Annex 4 of the Prevention and Control Plan Coronavirus Disease 2019 (Fifth Edition). COVID-19: Laboratory Testing Guideline [cited 2021 Jul 1]. http://www.chinacdc.cn/en/COVID19/202003/P020200308322036088669.pdf

8. Candido DS, Claro IM, de Jesus JG, Souza WM, Moreira FRR, Dellicour S, et al.; Brazil-UK Centre for Arbovirus Discovery, Diagnosis, Genomics and Epidemiology (CADDE) Genomic Network. Evolution and epidemic spread of SARS-CoV-2 in Brazil. Science. 2020;369:1255–60. PubMed https://doi.org/10.1126/science.abd2161

9. Larremore DB, Fosdick BK, Bubar KM, Zhang S, Kissler SM, Metcalf CJE, et al. Estimating SARS-CoV-2 seroprevalence and epidemiological parameters with uncertainty from serological surveys. eLife. 2021;10:e64206. PubMed https://doi.org/10.7554/eLife.64206

10. Harris PA, Taylor R, Thielke R, Payne J, Gonzalez N, Conde JG. Research electronic data capture (REDCap)—a metadata-driven methodology and workflow process for providing translational research informatics support. J Biomed Inform. 2009;42:377–81. PubMed https://doi.org/10.1016/j.jbi.2008.08.010

11. Barros AJ, Hirakata VN. Alternatives for logistic regression in cross-sectional studies: an empirical comparison of models that directly estimate the prevalence ratio. BMC Med Res Methodol. 2003;3:21. PubMed https://doi.org/10.1186/1471-2288-3-21

12. Gomes MGM, Corder RM, King JG, et al. Individual variation in susceptibility or exposure to SARS-CoV-2 lowers the herd immunity threshold. medRxiv preprint 2020. doi: https://doi.org/10.1101/2020.04.27.20081893. https://doi.org/10.1101/2020.04.27.20081893

13. Bastos LS, Ranzani OT, Souza TML, Hamacher S, Bozza FA. COVID-19 hospital admissions: Brazil's first and second waves compared. Lancet Respir Med. 2021;9:e82–3. PubMed https://doi.org/10.1016/S2213-2600(21)00287-3

Appendix Table 1. Characteristics of new SARS-CoV-2 genome sequences from Mâncio Lima, Amazonian Brazil, April 2021

Sample #	Average read depth (×)	Wuhan-Hu 1 genome coverage with 20× depth	Number of reads mapped	Bases covered >10×	Bases covered >25×	Pangolin lineage	Nextstrain clade
17	316	81%	20,387	26,883	24,194	P.1	20J (Gamma, V3)
13	329	79%	15,937	25,952	23,860	P.1	20J (Gamma, V3)
16	284	89%	35,228	28,341	27,451	P.1	20J (Gamma, V3)
22	117	95%	72,433	29,013	28,825	P.1	20J (Gamma, V3)
1	262	90%	34,923	28,234	27,237	P.1	20J (Gamma, V3)
38	83	53%	8,945	19,996	15,573	P.1	20J (Gamma, V3)
44	106	90%	45,431	28,073	27,394	P.1	20J (Gamma, V3)
39	365	94%	49,869	28,932	28,494	P.1	20J (Gamma, V3)
15	278	88%	46,836	28,542	27152	P.1	20J (Gamma, V3)
28	259	55%	7,492	19,600	18,295	P.1	20J (Gamma, V3)
23	257	81%	39,176	26,937	24,983	P.1.1	20J (Gamma, V3)

Appendix Table 2. Predictors of SARS-CoV-2 infection during the first and second epidemic waves in the Mâncio Lima cohort, Amazonian Brazil

	Models for the 2020 serosurvey							Models for the 2021 serosurvey						
	Unadjusted (n = 1,027)*				Adjusted (n = 1,027)†			Unadjusted (n = 729)*				Adjusted (n = 729)†		
Covariates	n	RR	95% CI	P	RR	95% CI	P	n	RR	95% CI	P	RR	95% CI	P
Individual level														
Age group														
0–5	55	1.00	Reference		1.00	Reference		44	1.00	Reference	.	1.00	Reference	.
6–15	235	0.89	0.62, 1.30	0.570	0.89	0.64, 1.26	0.531	166	0.98	0.59, 1.64	0.948	0.99	0.63, 1.56	0.975
16–30	222	0.94	0.65, 1.37	0.761	0.96	0.70, 1.31	0.783	183	1.17	0.71, 1.92	0.542	1.12	0.73, 1.73	0.596
31–49	307	0.78	0.54, 1.14	0.205	0.79	0.56, 1.12	0.185	235	1.09	0.66, 1.79	0.744	1.04	0.67, 1.61	0.854
≥50	208	0.76	0.51, 1.15	0.196	0.79	0.53, 1.16	0.220	101	0.39	0.19, 0.81	0.011	0.38	0.18, 0.77	0.008
Sex														
Female	572	1.00	Reference		1.00	Reference		395	1.00	Reference		1.00	Reference	
Male	455	0.85	0.71, 1.00	0.054	0.86	0.74, 0.99	0.031	334	0.96	0.76, 1.20	0.696	0.95	0.76, 1.17	0.609
Recent malaria														
No	961	1.00	Reference					709	1.00	Reference		1.00	Reference	
Yes	63	0.99	0.70, 1.41	0.966				18	1.52	0.86, 2.69	0.145	1.56	0.98, 2.47	0.059
Overnight out of town														
No	767	1.00	Reference		1.00	Reference		527	1.00	Reference		1.00	Reference	
Yes	256	1.14	0.95, 1.37	0.176	1.11	0.89, 1.38	0.375	200	1.27	1.00, 1.62	0.052	1.28	0.96, 1.70	0.091
Past dengue														
No	650	1.00	Reference		1.00	Reference		433	1.00	Reference				
Yes	377	1.14	0.96, 1.36	0.149	1.12	0.93, 1.35	0.222	296	0.94	0.74, 1.21	0.638			
Household level														
Wealth index quintile														
1 (poorest)	198	1.00	Reference		1.00	Reference		164	1.00	Reference		1.00	Reference	
2	197	1.20	0.91, 1.59	0.199	1.21	0.79, 1.87	0.384	154	1.05	0.76, 1.45	0.760	1.09	0.66, 1.79	0.744
3	206	1.40	1.03, 1.91	0.029	1.41	0.91, 2.19	0.128	143	0.82	0.56, 1.19	0.301	0.85	0.52, 1.41	0.539
4	211	1.17	0.86, 1.58	0.313	1.17	0.73, 1.86	0.513	142	0.97	0.67, 1.41	0.893	1.01	0.62, 1.63	0.971
5 (most affluent)	215	1.62	1.20, 2.17	0.001	1.60	1.04, 2.46	0.034	126	1.18	0.81, 1.73	0.384	1.25	0.74, 2.10	0.398
Household size														
1–3	375	1.00	Reference		1.00	Reference		269	1.00	Reference		1.00	Reference	
4–6	499	1.37	1.11, 1.70	0.004	1.38	1.04, 1.82	0.025	372	1.20	0.91, 1.59	0.199	1.20	0.84, 1.72	0.313
≥7	153	1.83	1.38, 2.41	<0.0001	1.87	1.23, 2.85	0.004	88	1.67	1.17, 2.39	0.005	1.68	1.00, 2.82	0.048
AIC		1483.2			1468.4				944.2			938.8		

Abbreviations: AIC, Akaike information criterion; CI, confidence interval; and RR, relative risk.
"Past dengue" refers to dengue fever seropositivity in the previous survey (2019 serology for 2020 models and 2020 serology for 2021 models).
*Totals may var.y for some covariates due to missing data.
†The adjusted model corresponds to the following STATA syntax*: mepoisson outcome indevars housevars || household: vce(robust) irr*. Relative risks are calculated for individual (*indevars*) and household-level covariates (*housevars*) included in the fixed-effects component.

Appendix Table 3. Predictors of clinically apparent COVID-19 during the first and second epidemic waves in the Mâncio Lima cohort, Amazonian Brazil.

	Models for the 2020 serosurvey							Models for the 2021 serosurvey						
	Unadjusted (n = 1,027)*				Adjusted (n = 1,027)†			Unadjusted (n = 729)*				Adjusted (n = 729)†		
Covariates	n	RR	95% CI	*P*	RR	95% CI	*P*	n	RR	95% CI	*P*	RR	95% CI	*P*
Individual level														
Age group														
0–5	55	1.00	Reference	.	1.00	Reference	.	44	1.00	Reference	.	1.00	Reference	.
6–15	235	1.16	0.47, 2.84	0.744	1.14	0.47, 2.74	0.771	166	1.57	0.37, 6.73	0.543	1.44	0.44, 4.75	0.548
16–30	222	2.31	0.97, 5.53	0.060	2.24	0.97, 5.22	0.060	183	4.52	1.16, 17.68	0.030	3.88	1.04, 14.48	0.044
31–49	307	2.07	0.87, 4.95	0.102	1.97	0.86, 4.54	0.111	235	4.43	1.13, 17.36	0.033	3.98	1.15, 13.78	0.029
≥50	208	2.31	0.94, 5.64	0.067	2.40	1.02, 5.68	0.046	101	1.46	0.30, 6.97	0.638	1.29	0.27, 6.10	0.744
Sex														
Female	572	1.00	Reference		1.00	Reference		395	1.00	Reference		1.00	Reference	
Male	455	0.87	0.68, 1.11	0.253	0.86	0.71, 1.04	0.127	334	0.87	0.60, 1.25	0.451	0.87	0.62, 1.23	0.427
Recent malaria														
No	961	1.00	Reference					709	1.00	Reference				
Yes	63	1.21	0.76, 1.92	0.431				18	1.27	0.44, 3.68	0.654			
Overnight out of town														
No	767	1.00	Reference					527	1.00	Reference		1.00	Reference	
Yes	256	1.06	0.81, 1.38	0.683				200	1.30	0.88, 1.91	0.189	1.29	0.80, 2.08	0.292
Past dengue														
No	650	1.00	Reference		1.00	Reference		433	1.00	Reference		1.00	Reference	
Yes	377	1.25	0.98, 1.61	0.074	1.31	1.00, 1.72	0.050	296	0.73	0.49, 1.08	0.117	0.75	0.49, 1.13	0.171
Household level														
Wealth index quintile														
1 (poorest)	198	1.00	Reference		1.00	Reference		164	1.00	Reference		1.00	Reference	
2	197	1.32	0.84, 2.10	0.231	1.26	0.65, 2.47	0.492	154	1.40	0.80, 2.45	0.245	1.32	0.58, 3.01	0.508
3	206	1.88	1.15, 3.05	0.011	1.85	1.00, 3.43	0.050	143	0.78	0.40, 1.54	0.475	0.82	0.36, 1.87	0.636
4	211	1.30	0.80, 2.11	0.283	1.24	0.64, 2.40	0.523	142	1.23	0.68, 2.22	0.500	1.26	0.58, 2.74	0.565
5 (most affluent)	215	2.48	1.57, 3.92	0.000	2.37	1.29, 4.35	0.005	126	1.66	0.89, 3.11	0.110	1.74	0.77, 3.95	0.183
Household size														
1–3	375	1.00	Reference		1.00	Reference		269	1.00	Reference		1.00	Reference	
4–6	499	1.63	1.21, 2.20	0.001	1.67	1.13, 2.47	0.010	372	1.37	0.91, 2.07	0.136	1.36	0.83, 2.25	0.224
≥7	153	2.58	1.74, 3.83	0.000	2.72	1.54, 4.80	0.001	88	0.47	0.17, 1.29	0.143	0.49	0.14, 1.69	0.259
AIC		1,061.5			1,036.9				580.4			573.3		

Abbreviations: AIC, Akaike information criterion; CI, confidence interval; and RR, relative risk.
"Past dengue" refers to dengue fever seropositivity in the previous survey (2019 serology for 2020 models and 2020 serology for 2021 models).
*Totals may var.y for some covariates due to missing data.
†The adjusted model corresponds to the following STATA syntax*: mepoisson outcome indevars housevars* || *household: vce(robust) irr*. Relative risks are calculated for individual (*indevars*) and household-level covariates (*housevars*) included in the fixed-effects component.

Appendix Table 4. Predictors of clinically manifest COVID-19 upon SARS-CoV-2 infection during the first and second epidemic waves.

	Models for the 2020 serosurvey							Models for the 2021 serosurvey						
	Unadjusted (n = 359)*				Adjusted (n = 359)†			Unadjusted (n = 209)*				Adjusted (n = 209)†		
Covariates	n	RR	95% CI	P	RR	95% CI	*P*	n	RR	95% CI	*P*	RR	95% CI	*P*
Individual level														
Age group														
0–5	21	1.00	Reference	.	1.00	Reference	.	13	1.00	Reference	.	1.00	Reference	.
6–15	88	1.28	0.57, 2.89	0.545	1.29	0.59, 2.81	0.518	50	1.40	0.38, 5.12	0.613	1.35	0.42, 4.35	0.615
16–30	87	2.47	1.14, 5.36	0.022	2.45	1.15, 5.22	0.020	62	3.36	1.00, 11.33	0.050	3.30	0.97, 11.27	0.057
31–49	100	2.55	1.17, 5.54	0.018	2.57	1.22, 5.41	0.013	73	3.75	1.10, 12.74	0.034	3.73	1.11, 12.54	0.034
≥50	63	2.83	1.30, 6.20	0.009	2.87	1.35, 6.08	0.006	11	3.51	0.95, 13.00	0.060	3.26	0.84, 12.58	0.087
Sex														
Female	214	1.00	Reference		1.00	Reference		116	1.00	Reference		1.00	Reference	
Male	145	0.99	0.84, 1.18	0.941	1.00	0.85, 1.17	0.999	93	1.03	0.79, 1.35	0.828	1.03	0.80, 1.31	0.835
Recent malaria														
No	336	1.00	Reference		1.00	Reference		200	1.00	Reference				
Yes	22	1.23	0.91, 1.66	0.181	1.22	0.92, 1.63	0.173	8	0.70	0.31, 1.59	0.393			
Overnight out of town														
No	256	1.00	Reference					143	1.00	Reference				
Yes	102	0.96	0.80, 1.14	0.626				66	1.03	0.78, 1.37	0.814			
Past dengue														
No	222	1.00	Reference		1.00	Reference		132	1.00	Reference		1.00	Reference	
Yes	137	1.16	0.98, 1.38	0.084	1.17	0.97, 1.41	0.096	77	0.81	0.60, 1.10	0.183	0.81	0.58, 1.12	0.198
Household level														
Wealth index quintile														
1 (poorest)	64	1.00	Reference		1.00	Reference		54	1.00	Reference		1.00	Reference	
2	71	1.11	0.79, 1.56	0.535	1.11	0.74, 1.67	0.624	48	1.03	0.68, 1.57	0.888	1.03	0.58, 1.86	0.911
3	76	1.30	0.93, 1.82	0.122	1.29	0.89, 1.87	0.185	33	0.70	0.41, 1.22	0.211	0.72	0.38, 1.34	0.299
4	65	1.15	0.82, 1.61	0.429	1.14	0.76, 1.69	0.528	38	1.03	0.66, 1.61	0.905	1.03	0.59, 1.80	0.920
5 (most affluent)	83	1.54	1.13, 2.11	0.007	1.51	1.07, 2.14	0.020	36	1.22	0.75, 1.98	0.416	1.24	0.68, 2.27	0.477
Household size														
1–3	107	1.00	Reference		1.00	Reference		64	1.00	Reference		1.00	Reference	
4–6	185	1.12	0.93, 1.36	0.238	1.14	0.91, 1.41	0.252	110	1.26	0.92, 1.72	0.151	1.24	0.88, 1.75	0.218
≥7	67	1.32	1.03, 1.69	0.029	1.33	0.99, 1.77	0.055	35	0.29	0.11, 0.78	0.013	0.29	0.09, 0.91	0.034
AIC		645.6			640.9				344.4			339.5		

Abbreviations: AIC, Akaike information criterion; CI, confidence interval; and RR, relative risk.
"Past dengue" refers to dengue fever seropositivity in the previous survey (2019 serology for 2020 models and 2020 serology for 2021 models).
*Totals may vary for some covariates due to missing data.
†The adjusted model corresponds to the following STATA syntax*: mepoisson outcome indevars housevars* || *household: vce(robust) irr*. Relative risks are calculated for individual (*indevars*) and household-level covariates (*housevars*) included in the fixed-effects component.

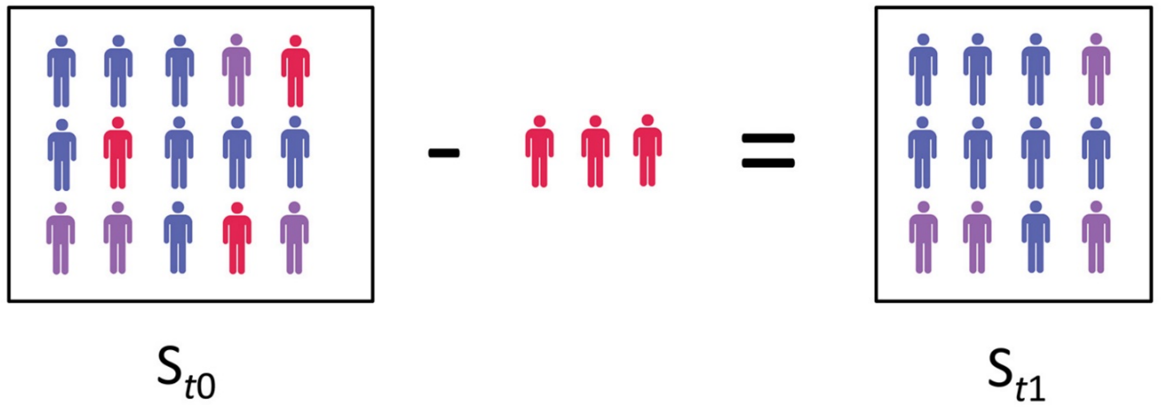

Appendix Figure 1. Selective removal of high-risk cohort participants from the seronegative (= fully susceptive) pool as the COVID-19 pandemic develops. We represent a population with heterogeneous risk of infection (from blue to red) from which high-risk individuals (red) are selectively removed by infection, from the fully susceptible population S, between times t_0 and t_1. This decreases the average susceptibility to infection in the cohort of seronegatives left behind. As a consequence, the rate of new seroconversions tends to decrease unless viral transmissibility increases over time. if this selection process also affects the susceptibility to disease upon infection, the proportion of infections leading to clinical manifestations and severe disease may also decrease with time unless more virulent virus variants are introduced in the population.

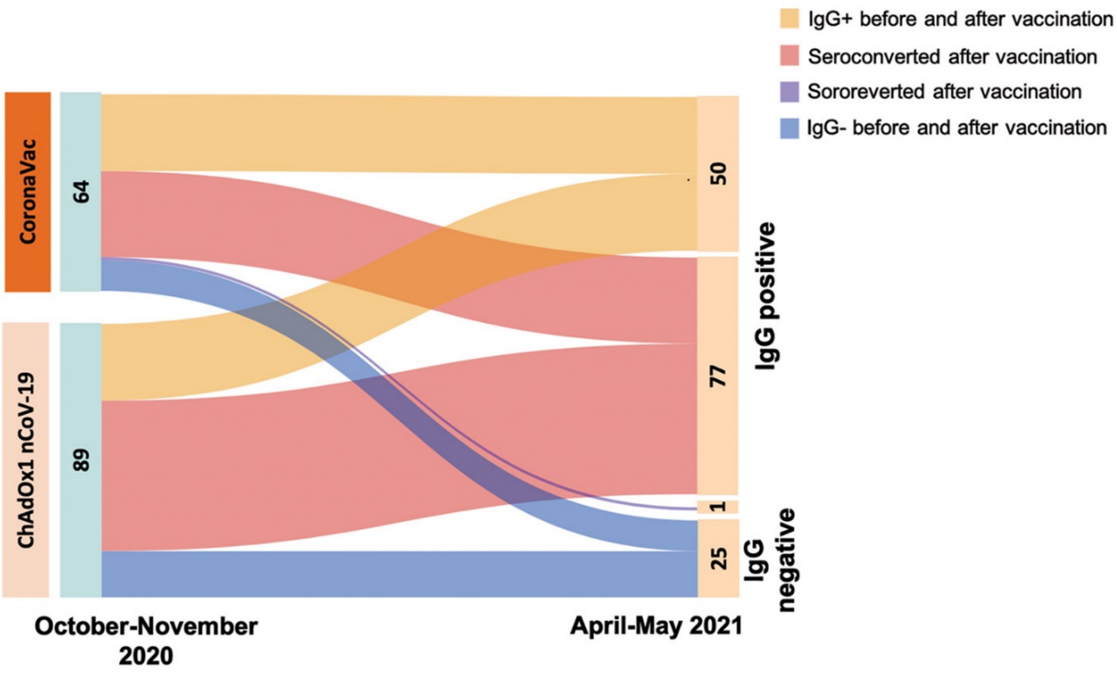

Appendix Figure 2. IgG antibody responses to SARS-CoV-2 in vaccinated participants.

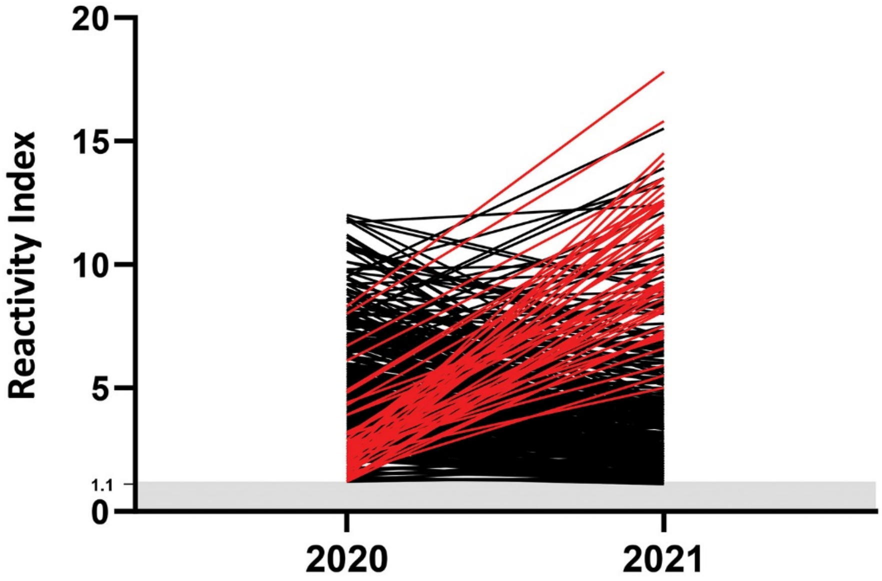

Appendix Figure 3. Paired IgG antibody reactivity indices to SARS-CoV-2 in 347 study participants who remained seropositive from October-November 2020 to April-May 2021. Results for 46 study participants who had a >2-fold increase in reactivity indices are shown in red.

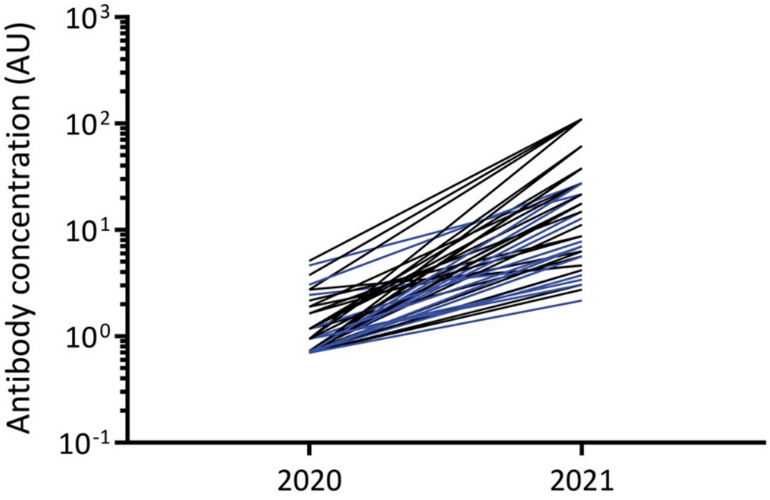

Appendix Figure 4. Paired SARS-CoV-2 IgG concentrations (in arbitrary units, AU) in 46 study participants who had a >2-fold increase in reactivity indices between October-November 2020 and April-May 2021. Results for 18 unvaccinated study participants are shown in blue.
